# Older Candidates for Subthalamic Deep Brain Stimulation in Parkinson's Disease Have a Higher Incidence of Psychiatric Serious Adverse Events

**DOI:** 10.3389/fnagi.2016.00132

**Published:** 2016-06-08

**Authors:** Vitalii V. Cozac, Michael M. Ehrensperger, Ute Gschwandtner, Florian Hatz, Antonia Meyer, Andreas U. Monsch, Michael Schuepbach, Ethan Taub, Peter Fuhr

**Affiliations:** ^1^Department of Neurology, Hospital of the University of BaselBasel, Switzerland; ^2^Memory Clinic, Felix Platter Hospital, University Center for Medicine of AgingBasel, Switzerland; ^3^Department of Neurology, University Hospital Bern and University of BernBern, Switzerland; ^4^Département de Neurologie, Assistance-Publique Hôpitaux de Paris, Centre d'Investigation Clinique 9503, Institut du Cerveau et de la Moelle épinière, Université Pierre et Marie Curie, Paris 6 et Institut National de la Santé et de la Recherche Médicale, Centre Hospitalier Universitaire (CHU) Pitié-SalpêtrièreParis, France

**Keywords:** deep brain stimulation (DBS), Parkinson's disease (PD), psychosis, adverse events, age factors

## Abstract

**Objective:** To investigate the incidence of serious adverse events (SAE) of subthalamic deep brain stimulation (STN-DBS) in elderly patients with Parkinson's disease (PD).

**Methods:** We investigated a group of 26 patients with PD who underwent STN-DBS at mean age 63.2 ± 3.3 years. The operated patients from the EARLYSTIM study (mean age 52.9 ± 6.6) were used as a comparison group. Incidences of SAE were compared between these groups.

**Results:** A higher incidence of psychosis and hallucinations was found in these elderly patients compared to the younger patients in the EARLYSTIM study (*p* < 0.01).

**Conclusions:** The higher incidence of STN-DBS-related psychiatric complications underscores the need for comprehensive psychiatric pre- and postoperative assessment in older DBS candidates. However, these psychiatric SAE were transient, and the benefits of DBS clearly outweighed its adverse effects.

## Introduction

Deep brain stimulation (DBS) is widely used as a neurosurgical treatment for Parkinson's disease (PD), because it improves the motor manifestations of PD and reduces the need for antiparkinsonian medication (Fasano and Lozano, 2015). The intraoperative, short-term, and long-term adverse effects of DBS in PD are well-known (Falowski et al., 2015). However, when candidates for DBS are appropriately selected, the benefit of the procedure in terms of an improved quality of life generally justifies its small risk. Moreover, it was concluded in the EARLYSTIM study (Schuepbach et al., 2013) that DBS in the subthalamic nucleus (STN-DBS) yielded a better outcome than drug treatment alone in patients with early (rather than advanced) motor complications of PD. In that study, the incidence of serious adverse events (SAE) was compared in patients in the medical versus the surgical arms of the study. The mean age of the 124 patients who underwent surgery was 52.9 years, and they were followed up for 2 years. Today, no consensus exists regarding an age cut-off for DBS as a treatment of PD (Vesper et al., 2007; Floden et al., 2014). In the present study, we retrospectively determined the incidence of SAE after STN-DBS in a group of patients whose mean age was 63.2 years and compared it to the incidence of SAE among the patients in the EARLYSTIM study.

## Methods

### Patients

We retrospectively analyzed the medical records of PD patients who underwent STN-DBS in our institution, which were extracted from the clinical and research databases of the University Hospital Basel. The study protocol was approved by the local ethics committee (Ethikkommission beider Basel). The records were analyzed for a period of 2 years after STN-DBS. The inclusion and exclusion criteria from the EARLYSTIM study (Schuepbach et al., 2013) were used to select cases for the analysis, with the exception of age: in the present study we focused on a group of relatively old operated patients (see Supplement 1 for the criteria of selection). The age of the patients in the EARLYSTIM study ranged from 18 to 60 years; in the group of patients from our database, the age ranged from 58 to 70 years.

Twenty-six patients with PD (11 women, 15 men) who underwent STN-DBS from January 1, 2008 to June 30, 2013, were selected for the analysis (i.e., the “BASEL group” of patients in what follows, as opposed to the “EARLYSTIM group”).

### Operative procedure for STN-DBS

In each patient, Medtronic 3389 electrodes were stereotactically implanted into the STN bilaterally under local anesthesia with the aid of intraoperative microelectrode recording and test stimulation, after coordinate- and visually-based target selection and trajectory calculation with the aid of preoperative CT and MRI scans. The pulse generator was implanted subsequently under general anesthesia.

### Analyses of the cases

Patients with PD who were scheduled for DBS underwent interdisciplinary assessment (including detailed neurological and neuropsychological examinations) before and, in general, every 6 months after the procedure. The results of these assessments were stored in the hospital's clinical and research databases and were compiled for this analysis. Whenever a patient needed his/her family doctor's assistance for problems potentially related to Parkinson's disease or to DBS, but was not admitted to the hospital for these problems, the family doctor reported such cases to the clinical and research database. The interdisciplinary assessment performed within the 14 days before the STN-DBS will be referred to as the “baseline assessment,” while that performed 24 months later will be referred to as the “2-year assessment.”

The following variables were analyzed: subscales II and III of the Unified Parkinson's Disease Rating Scale (UPDRS), the Brief Psychiatric Rating Scale (BPRS), the Beck Depression Inventory II (BDI II), and the levodopa equivalent daily dose (LEDD). In some cases, diagnoses made by other medical specialists (e.g., a cardiologist) were used in addition as an indication of the patient's general medical condition.

SAE were defined according to the Medical Dictionary for Regulatory Activities, version 14.1, as any registered events that led to death, disability, or prolonged or new hospitalization with serious health impairment. The list of SAE was adjusted to the list of SAE described in the EARLYSTIM study. Thus, the SAE were grouped in the following categories: (1) suicide, (2) life-threatening events, (3) events related to medication or stimulation, (4) events related to surgery or device, (5) events related to PD. We calculated the number of reported SAE in each category for each patient within the 24 months after STN-DBS.

### Statistical analysis

Statistical calculations were done with the R version 3.1.2 open source software. Baseline mean and standard deviations of the demographic and clinical parameters of the groups were compared with an unpaired Student's *t*-test. The numbers of patients with SAE were compared with the chi-squared test with Yates correction to prevent overestimation of statistical significance for small data; significance was set at *p* < 0.05. Linear regression models were applied to adjust for potential confounders in statistically significant differences.

## Results

The results of the comparison of the demographic and clinical features of the two groups are shown in Supplement 2. The patients in the BASEL group were older than the patients in the EARLYSTIM study who underwent surgery (mean difference 10.3 years, 95% confidence interval (CI) 7.7 to 12.9). Disease duration in the BASEL group was longer (mean difference 2.7 years, CI: 1.3 to 4.1).

The results of the comparison of SAE incidence between the two groups are shown in Table 1. Significant differences in the incidence of SAE were found in the category “Event related to medication or stimulation” (Chi-squared = 4.5, *p* = 0.03) and in its subcategory “psychosis and hallucinations” (Chi-squared = 24.7, *p* < 0.01). The characteristics of psychosis and hallucination of the patients in the BASEL group are shown in Table 2. Regression models showed no influence of clinical and demographic parameters on the incidence of psychosis.

**Table 1:** **Serious adverse events in the first 2 years after STN-DBS surgery**.

**Parameters**	**EARLYSTIM STN-DBS (*****n*** = **124)**		**BASEL group (*****n*** = **26)**		**Chi-square test, *p*-value**
**Event**	**No. of events**	**No. of patients (%)**	**No. of events**	**No. of patients (%)**	
Total serious adverse events	123	68 (54.8)	61	18 (69.2)	ns
1. Death, all by suicide	2	2 (1.6)	0	0	ns
2. Life-threatening event	14	12 (9.7)	2	2 (7.7)	ns
3. Event related to medication or stimulation	24	24 (19.4)	10	10 (38.5)	0.03
Worsening of mobility^*^	5	5 (4.0)	1	1 (3.8)	ns
Motor fluctuations	0	0	1	1 (3.8)	ns
Dyskinesia	1	1 (0.8)	0	0	ns
Psychosis or hallucinations	0	0	5	5 (19.2)	< 0.01
Anxiety	0	0	0	0	–
Impulse control disorder	1	1 (0.8)	0	0	ns
Depression	6	6 (4.8)	2	2 (7.7)	ns
Suicidal ideation	1	1 (0.8)	0	0	ns
Suicidal attempt	2	2 (1.6)	0	0	ns
Cardiac disorder	0	0	0	0	–
Injury	3	3 (2.4)	0	0	ns
Respiratory or thoracic disorder	1	1 (0.8)	0	0	ns
Other	4	4 (3.2)	0	0	ns
4. Event related to surgery or device	26	22 (17.7)	8	8 (30.8)	ns
Impaired wound healing	4	4 (3.2)	2	2 (7.7)	ns
Intracerebral abscess or edema	2	2 (1.6)	2	2 (7.7)	ns
Dislocation of device^**^	5	4 (3.2)	1	1 (3.8)	ns
Reoperation necessary^***^	4	2 (1.6)	2	2 (7.7)	ns
Other	11	10 (8.1)	1	1 (3.8)	ns
5. Event related to PD	57	39 (31.5)	41	10 (38.5)	ns

* *Worsening of mobility was defined as tremor, rigidity, akinesia, wearing off of medication effect, dystonia, or worsening of symptoms of PD*.

** *Dislocation of device was defined as dislocation of the stimulator, cable, or lead*.

*** *Reoperation was necessary in order to repair the stimulator or lead*.

**Table 2:** **Patients with serious psychosis and hallucinations**.

**No**	**Age (years)**	**Sex**	**Clinical presentation**	**Time of manifest after STN-DBS**	**Result**
1	60	Female	Visual illusions, sense of presence, fear	8 weeks	New hospitalization
2	62	Female	Delusion of spousal infidelity, suspicions of harmful thoughts	8 weeks	New hospitalization
3	60	Male	Passage hallucinations, delusion	16 weeks	New hospitalization
4	69	Male	Paranoia with aggression	10 days	Prolonged hospitalization
5	63	Female	Visual illusions, sense of presence, fear	12 weeks	New hospitalization

## Discussion

We found a higher incidence of psychosis and hallucinations after STN-DBS in a sample of PD patients who were about 10 years older than those of the EARLYSTIM study.

The effects of DBS on mental functioning are not clear, and the pattern and expression of neuropsychiatric symptoms in operated patients with PD are highly variable (Volkmann et al., 2010). Some researchers have reported various types of psychiatric side effects of DBS, ranging from apathy and emotional lability to visual hallucinations, hypersexuality, and aggressive behavior (Soulas et al., 2008; Le Jeune et al., 2009; Bickel et al., 2010; Daniele et al., 2012; Qureshi et al., 2015). In a meta-analysis of 808 publications covering the years 1996–2005, the most common psychiatric side effect associated with DBS was delirium, making up 4 to 8% of all psychiatric complications (Appleby et al., 2007). The psychiatric side effects of DBS are usually transient and treatable (Voon et al., 2006), but there have been case reports of very severe side effects with long-term consequences (Zonana et al., 2011; Piccoli et al., 2015). Other researchers reported an improvement of psychiatric symptoms after DBS (Funkiewiez et al., 2004). Vesper et al. analyzed the outcomes of DBS in PD as a function of age (Vesper et al., 2007), comparing patients over and under age 65 1 year after surgery. Transient neuropsychiatric impairment was a common finding in both groups (the adverse events were not stratified with regard to severity). In another observational study by Shiina et al. a sample of PD patients with a mean age of 65 years was followed up for 12 months after DBS (Shiina et al., 2015): some experienced an improvement of pre-operative psychiatric symptoms, some had psychiatric side effects, and others had no changes in psychiatric state. Piasecki et al. proposed three possible mechanisms of mental disturbance after DBS: effects of the electrode placement itself, neurotransmitter changes induced by stimulation, and worsening of a pre-existing mental disorder by DBS (Piasecki and Jefferson, 2004). Three regions have been described in the STN in which the neurons participate in the following functional circuits: sensorimotor (dorsolateral), motor (ventromedial), and limbic (medial) (Romanelli et al., 2004). Limbic circuits play a role in emotional and behavioral control. Spread of electrical current from the subthalamic DBS electrodes may cause a disturbance in the limbic circuit, producing psychiatric symptoms. Another explanatory hypothesis for psychiatric complications after DBS is based on an imbalance of two neuromodulators, γ-aminobutyric acid (GABA), and glutamate. DBS leads to an increased activity of nigral GABAergic neurons and decreased activity of glutamate flow from the STN (Malhi and Sachdev, 2002; Piasecki and Jefferson, 2004). Dysfunction of these transmitters is known to be involved in psychiatric disorders (Drevets et al., 1997). Another possible explanation of post-DBS psychosis is the reduction or withdrawal of dopaminergic medication after surgery (Voon et al., 2006). Finally, some researchers have seen a relation between post-DBS psychosis and pre-existing mental disturbances and have therefore stressed the importance of a thorough psychiatric assessment as a prerequisite for DBS surgery (Kalteis et al., 2006). We hypothesize that the psychiatric SAE in our study were of multifactorial origin and that their higher incidence in older patients may be explained by these patients' lower resistance to surgical stress and their decreased neuroplasticity, resulting in a poorer ability to reset the functional limbic circuit affected by PD and DBS (Saint-Cyr et al., 2000).

It should be noted that the STN is not the only possible target for DBS in the treatment of PD. In particular, DBS in the internal segment of the globus pallidus (GPi) has been found to be comparable to STN-DBS in terms of efficacy and safety (Honey et al., 2016). However, GPi-DBS has its own benefits and limitations (Groiss et al., 2009). Some centers favor the pallidal target for certain groups of patients (Okun et al., 2009).

Our analysis has several limitations. First, the patients from the BASEL group were not very old, but still within the generally accepted age limits for the operation (Okun and Foote, 2010). Second, while the EARLYSTIM data were obtained in a multicenter trial, the findings we report here are derived from a single center. Single-center analyses have a role in planning and powering of subsequent larger studies (Bellomo et al., 2009); thus, this report may serve as a starting point for further research into the age-dependent effects of DBS in PD. Finally, a detailed comparison of the cognitive performance of the patients in the two groups is impossible on the basis of the information available in the EARLYSTIM report.

In spite of these limitations, a comparison of these two groups enabled us to estimate the influence of age on the neuropsychiatric and neuropsychological outcome of STN-DBS surgery in patients with PD. The apparently higher incidence of psychiatric complications after STN-DBS in older patients underscores the need for comprehensive pre- and postoperative psychiatric assessment in older DBS patients. However, the psychiatric SAE that arose in our patients (the BASEL group) were transient, occurring mainly in the early postoperative period. The benefits of DBS clearly outweighed its adverse effects in this group of patients.

## Author contributions

VC: conceived and designed the study and was responsible for its execution, wrote the first draft, performed data analyses, contributed core ideas, and was involved in critically revising the manuscript; ME: supervised the neuropsychological evaluations, contributed core ideas, and was involved in critically revising the manuscript; UG: conceived and designed the study and was responsible for its execution, performed data analyses, was responsible for the psychiatric and psychological assessments, contributed core ideas, and was involved in critically revising the manuscript; FH: performed neurologic assessments, managed the database infrastructure, contributed core ideas, and was involved in critically revising the manuscript; AM: was responsible for the psychological assessments, contributed core ideas, and was involved in critically revising the manuscript; AUM: supervised the neuropsychological evaluations, contributed core ideas, and was involved in critically revising the manuscript; MS: contributed core ideas and was involved in critically revising the manuscript; ET: performed the DBS procedures, contributed core ideas, and was involved in critically revising the manuscript; PF: conceived and designed the study and was responsible for its execution, contributed core ideas, was involved in critically revising the manuscript.

## Funding

Supported by the Parkinson Schweiz, the Bangerter-Rhyner Foundation, the Gossweiler Foundation, the Freie Akademische Gesellschaft Basel, and the Camelia Botnar Foundation. These institutions had no further role in the study design or data evaluation and interpretation.

## Disclosures

VC: grant from the Camelia Botnar Foundation; ME: grant from Vifor Pharma; UG: grants from the Parkinson Schweiz, the Gossweiler Foundation, the Freiwillige Akademische Gesellschaft Basel, the Bangerter-Rhyner Foundation, the Swiss National Science Foundation, the Camelia Botnar Foundation, the Hedwig Widmer Foundation, unrestricted grants from: UCB Pharma AG, Abbvie AG, General Electrics; FH: grant from the Freiwillige Akademische Gesellschaft Basel; AM: grant from the Hedwig Widmer Foundation; AUM: grants from the Alzheimer Forum Switzerland, the Swiss Alzheimer's Association, Vifor Pharma, Advisory Boards: MSD, AC Immune, Takeda, Zinfandel Pharma; ET: grant from the Swiss National Science Foundation; MS: has nothing to declare; PF: grants from the Parkinson Schweiz, the Gossweiler Foundation, the Freiwillige Akademische Gesellschaft Basel, the Bangerter-Rhyner Foundation, the Swiss National Science Foundation, the Swiss Multiple Sclerosis Society, the Camelia Botnar Foundation, the Hedwig Widmer Foundation, unrestricted grants from: UCB Pharma AG, Roche AG, Abbvie AG, General Electrics, Advisory Board: Biogen Inc.

### Conflict of interest statement

The authors declare that the research was conducted in the absence of any commercial or financial relationships that could be construed as a potential conflict of interest.
