# Supplementary material for: Older Candidates for Subthalamic Deep Brain Stimulation in Parkinson's Disease Have a Higher Incidence of Psychiatric Serious Adverse Events
Source: Front Aging Neurosci. 2016 Jun 8;8:132. doi: 10.3389/fnagi.2016.00132 (PMC4896943; doi:10.3389/fnagi.2016.00132)
Supplement: Supplementary file 1 [file DataSheet1.DOCX]

**Supplement 1. Selection criteria for the cases**

The major inclusion criteria were adjusted to those, used in the EARLYSTIM study:

- diagnosis of idiopathic Parkinson’s disease with a duration of at least four years before deep brain stimulation of the subthalamic nuclei (STN-DBS);
- STN-DBS was performed before 1 July 2013;
- moderate disease severity (i.e. Hoehn and Yahr score less than 3 in the “ON” medication state, assessed within 14 days before the STN-DBS).

The exclusion criteria were:

- dementia according to Diagnostic and Statistical Manual of mental disorders version IV (DSM-IV);
- severe depression (i.e. a score of 25 or higher on the Beck Depression Inventory II);
- any psychotic disorder according to DSM-IV criteria.
