# Supplementary material for: Older Candidates for Subthalamic Deep Brain Stimulation in Parkinson's Disease Have a Higher Incidence of Psychiatric Serious Adverse Events
Source: Front Aging Neurosci. 2016 Jun 8;8:132. doi: 10.3389/fnagi.2016.00132 (PMC4896943; doi:10.3389/fnagi.2016.00132)
Supplement: Supplementary file 2 [file DataSheet2.docx]

**Supplement 2, Table 3.**

**Demographic and clinical features of the groups at baseline.**

| Variable | EARLYSTIM STN-DBS | BASEL group | t-test |
| --- | --- | --- | --- |
| No of subjects | 124 | 26 | - |
| Males (%) | 94 (75.8) | 15 (57.7) | ns |
| Age (mean ±SD) | 52.9 ±6.6 | 63.2 ±3.3 | p<0.01 |
| Duration of PD (mean ±SD) | 7.3 ±3.1 | 10.0 ±3.7 | p<0.01 |
| LEDD (mean ±SD) | 918.8 ±412.5 | 962.0 ±562.6 | ns |
| BPRS score (mean ±SD) | 25.3 ±1.0 | 24.8 ±5.1 | ns |
| BDI II score (mean ±SD)  “ON” medication state | 10.1 ±0.6 | 9.5 ±4.0 | ns |
| UPDRS II (mean ±SD) | 15.0 ±0.8 | 15.4 ±5.8 | ns |
| UPDRS III (mean ±SD) | 33.2 ±1.8 | 34.8 ±12.5 | ns |

LEDD – levodopa equivalent daily dose; BPRS – Brief Psychiatric Rating Scale; BDI II – Beck Depression Inventory II; UPDRS –Unified Parkinson Disease Rating Scale.
